# Angiotensin II Induced Cardiac Dysfunction on a Chip

**DOI:** 10.1371/journal.pone.0146415

**Published:** 2016-01-25

**Authors:** Renita E. Horton, Moran Yadid, Megan L. McCain, Sean P. Sheehy, Francesco S. Pasqualini, Sung-Jin Park, Alexander Cho, Patrick Campbell, Kevin Kit Parker

**Affiliations:** 1 Disease Biophysics Group, Wyss Institute for Biologically Inspired Engineering, John A. Paulson School of Engineering and Applied Sciences, Harvard University, Cambridge, Massachusetts, United States of America; 2 Department of Agriculture and Biological Engineering, James Worth Bagley College of Engineering, College of Agriculture and Life Sciences, Mississippi State University, Starkville, Mississippi, United States of America; Niigata University Graduate School of Medical and Dental Sciences, JAPAN

## Abstract

*In vitro* disease models offer the ability to study specific systemic features in isolation to better understand underlying mechanisms that lead to dysfunction. Here, we present a cardiac dysfunction model using angiotensin II (ANG II) to elicit pathological responses in a heart-on-a-chip platform that recapitulates native laminar cardiac tissue structure. Our platform, composed of arrays of muscular thin films (MTF), allows for functional comparisons of healthy and diseased tissues by tracking film deflections resulting from contracting tissues. To test our model, we measured gene expression profiles, morphological remodeling, calcium transients, and contractile stress generation in response to ANG II exposure and compared against previous experimental and clinical results. We found that ANG II induced pathological gene expression profiles including over-expression of natriuretic peptide B, Rho GTPase 1, and T-type calcium channels. ANG II exposure also increased proarrhythmic early after depolarization events and significantly reduced peak systolic stresses. Although ANG II has been shown to induce structural remodeling, we control tissue architecture via microcontact printing, and show pathological genetic profiles and functional impairment precede significant morphological changes. We assert that our *in vitro* model is a useful tool for evaluating tissue health and can serve as a platform for studying disease mechanisms and identifying novel therapeutics.

## Introduction

Cardiomyopathies activate a number of compensatory mechanisms that trigger genetic, structural, and functional remodeling in order to maintain cardiac output. In instances of chronic or severe overload, remodeling continues to progress towards more maladaptive remodeling culminating in heart failure. During maladaptive cardiac remodeling, the heart tissue becomes fibrotic [1, 2], the ventricle thickens or dilates [3] and cardiomyocytes undergo further shape changes [4–6]. Gene expression profiles also revert back to a fetal state, where genes such as T-box5, brain natriuretic factor, ion channels, and extracellular matrix related genes are upregulated [7]. These changes synergistically lead to functional impairment, such as alterations in excitation-contraction coupling, changes in calcium handling, and contractile dysfunction [8, 9].

ANG II, an octapeptide hormone of the renin angiotensin system, is produced in multiple organs including the kidney, liver, and heart [10], and is a key factor in cardiovascular homeostasis, fibrosis, hypertrophy, and heart failure [11, 12]. ANG II has two main receptors, AT_1_ receptor and AT_2_ receptor, which are expressed in varying abundances depending on cell type and cell age [13]. AT_1_ receptor plays a central role in cardiac pathogenesis, mediating extracellular remodeling, proliferation, inflammation, and hypertrophy [13]. The transiently expressed AT_2_ receptor [14] counteracts AT_1_ receptor effects by inducing apoptosis and inhibiting cell growth that accompanies hypertrophy [15]. *In vivo* studies in mice reveal that chronic endogenous exposure to ANG II leads to impaired cardiac function [16]. ANG II has been shown to contribute to contractile dysfunction in failing rat hearts [17] and elicits negative inotropic behavior in the form of decreased shortening amplitude and shortening velocity in neonatal rat cardiomyocytes [18]. Under pathological conditions, negative inotropic effects are accompanied by elevated cardiac ANG II levels and ANG II plasma levels [19]. These findings suggest that ANG II plays a pivotal role in cardiac dysfunction. A number of *in vitro* studies have probed the effects of ANG II on single or sparsely seeded cardiomyocytes [18, 20, 21]. We sought to build upon these studies by examining the acute direct effect of ANG II on anisotropic, laminar cell monolayers. For simplicity, we termed these multicellular constructs engineered cardiac tissues, for they mimic the architecture of a single myocardial layer. While chronic ANG II exposure has been linked to heart failure, acute ANG II studies can provide insight into initiating stressors that lead to disease onset. Here, we present our model as a tool that can be used to understand early signs of cardiac impairment which can manifest into disease.

We proposed to build a cardiac dysfunction model by exploiting the pathological effects of ANG II using engineered tissues composed of neonatal rat ventricular cardiomyocytes that mimic the laminar tissue structure of myocardium on our muscular thin film (MTF) chip. The goal of this study was to demonstrate that our system could effectively recapitulate cardiac dysfunction by testing the effects of ANG II on gene expression profiles, cell morphology, cytoskeletal organization, calcium transients, and contractile stress generation. We found that ANG II exposure induced pathophysiological genetic trends, increased occurrences of early after depolarization events, and elicited a negative inotropic effect in engineered cardiac tissues. Importantly, these pathophysiological properties manifested in the chip while cell structure was strictly controlled, suggesting cardiac dysfunction may precede structural remodeling. Based on our results, we assert that we can effectively mimic a cardiac pathophysiological effect *in vitro* by exposing engineered cardiac tissues to ANG II. Moreover, our findings support the use of organs on chips for disease modelling and tissue level mechanistic studies to better understand early stage disease development which has the potential to uncover novel therapeutic interventions to prevent disease progression.

## Results

### Cardiac tissue and model design

**Fig 1:**
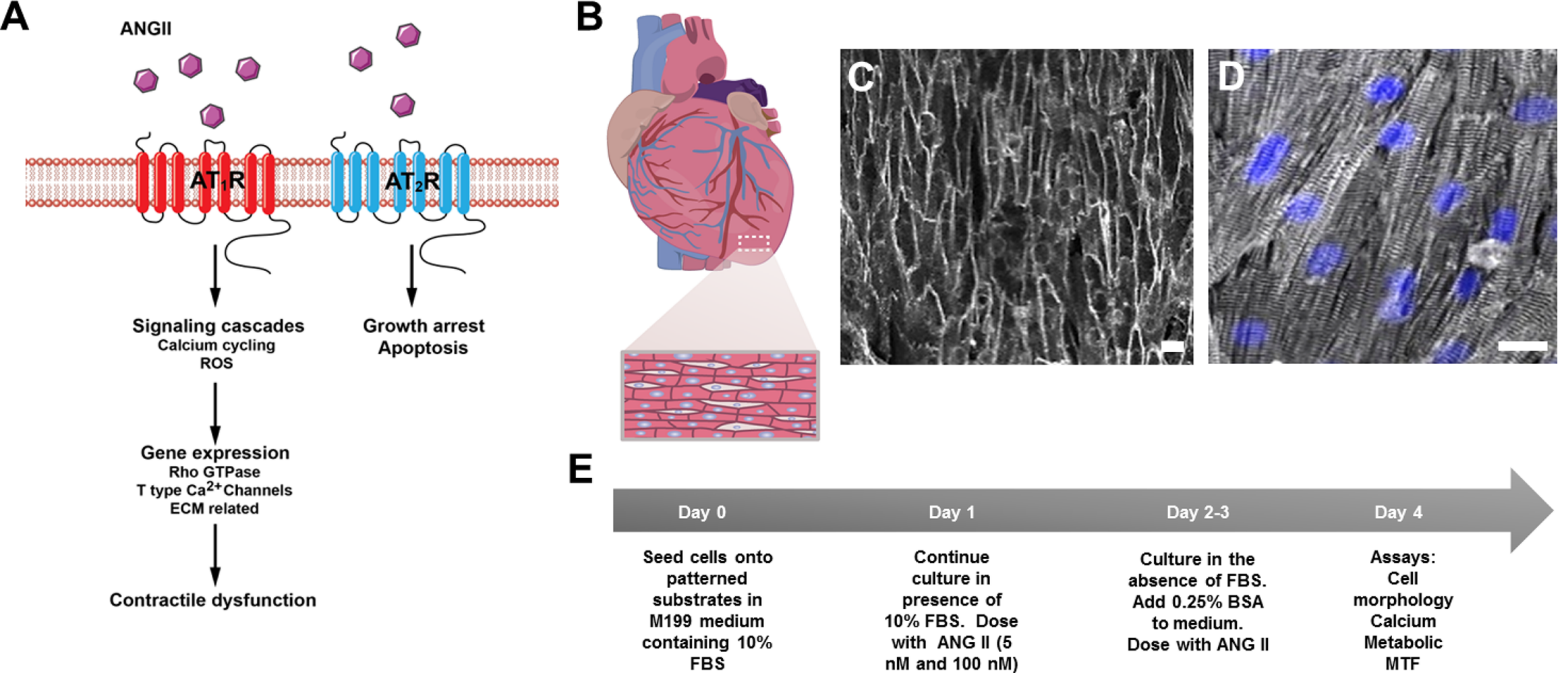
ANG II mechanism, native myocardium tissue architecture, engineered tissues, and experimental protocol. (A) ANG II mechanism. (B) Schematic of the heart depicting structural architecture of the ventricle which consists of aligned cardiomyocytes. (C) Engineered cardiac tissues were stained with di-8-ANEPPS (gray) to delineate the cell borders within the tissues for cell shape analysis. (D) Engineered cardiac tissues were stained for sarcomeric α-actinin (gray) and dapi (blue) and exhibit well aligned sarcomeres (Scale bars: 20 μm). (E) ANG II induced cardiac dysfunction experimental protocol.

We reasoned that we could stimulate neonatal rat ventricular cardiomyocytes with ANG II, a known hypertrophic stimulator that contributes to heart failure [20, 22, 23], to create cardiac dysfunction on a chip (Fig 1A). The native myocardium is composed of anisotropic, laminar tissue (Fig 1B). We employed methods detailed previously [24, 25] to build our cardiac dysfunction model composed of arrays of PDMS cantilevers, MTFs. The chips were UV ozone treated, stamped with 15μm wide fibronectin lines separated by 2 μm spaces, and background treated with fibronectin. Neonatal rat ventricular cardiomyocytes were seeded onto the chips to self-organize into anisotropic tissues that resembled the structure of native ventricular tissue (Fig 1C and 1D). Tissues were treated with ANG II (5 nM or 100 nM) every 24 h for the duration of the experiment (Fig 1E). Using our chips, we were able to assess the effects of ANG II on gene expression, structural remodeling, and tissue function.

### Angiotensin II induces pathological gene expression

**Fig 2:**
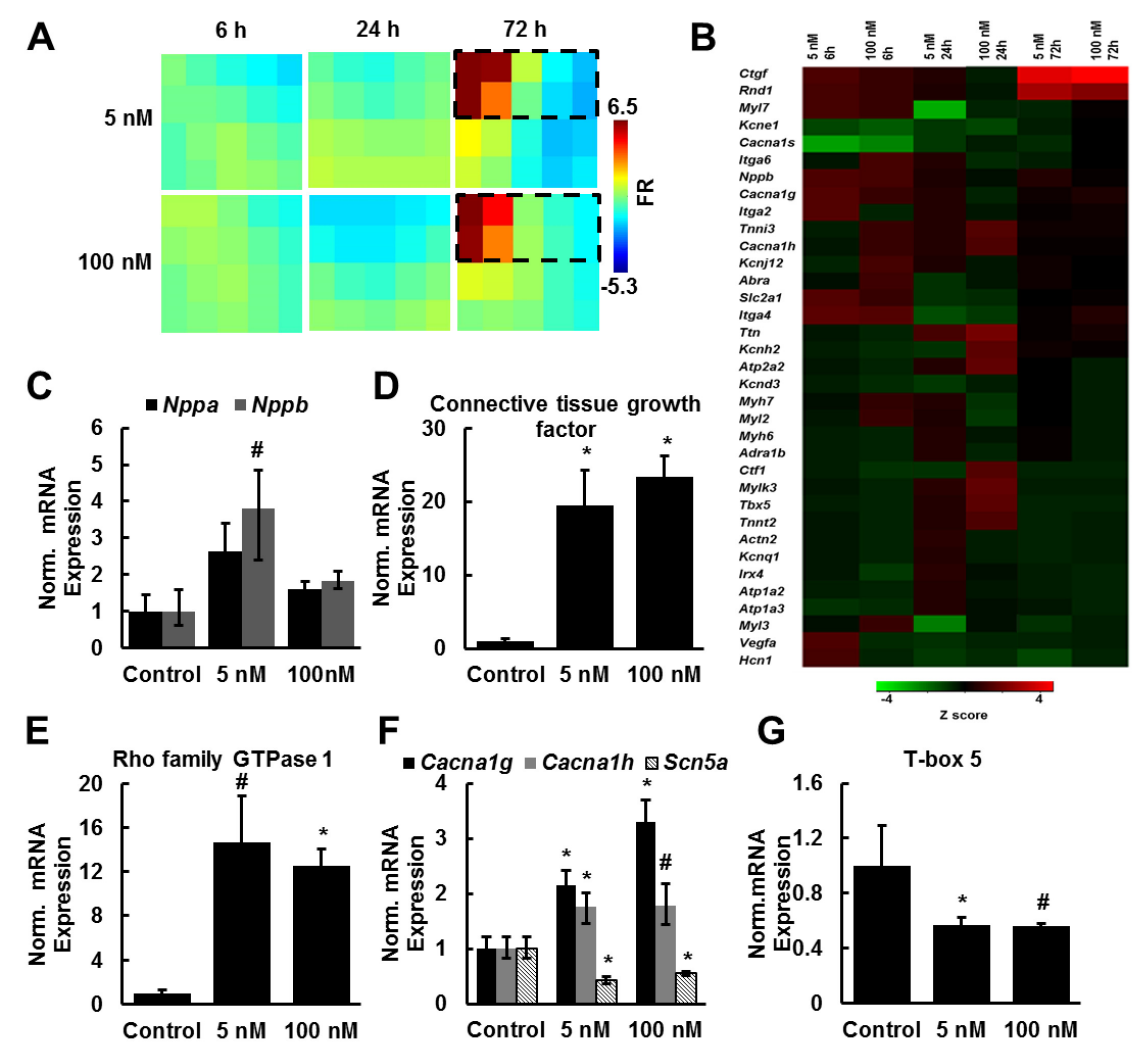
ANG II induces pathological gene expression profiles. (A) GEDI maps were created from qRT-PCR array fold regulation data over the duration of the experiment. Fold regulation (FR) was normalized to control tissues at the respective time point. Gene expression profiles were most different from the control at 72h of ANG II exposure. (B) Z scores were used to create a heatmap of genes of interest from the GEDI analysis (genes outlined in (A)). (C-G) Genes were significantly upregulated or downregulated in response to ANG II exposure (mean ± SEM, n = 3 tissues; # indicates p ≤ 0.01vs. control 72 h, * indicates p ≤ 0.05 vs. control 72 h).

We asked whether ANG II exposure would induce gene expression profiles characteristic of pathological hypertrophy and heart failure in our system. To measure relative gene expression in treated and untreated tissues, cell lysates were collected at 6, 24, and 72 h after the initial ANG II dose. Tissues were administered 5 nM or 100 nM ANG II every 24 h, ranges previously reported [18, 20]. Relative fold regulation and fold change were normalized to untreated control groups at respective time points. We performed qRT-PCR using Qiagen custom PCR arrays containing 86 genes known to be influenced by hypertrophy and/or heart failure (S1–S3 Tables) [26]. The Gene Expression Dynamic Inspector (GEDI) was then used to create a color coded mosaic composed of gene clusters grouped based on gene expression profile similarities (Fig 2A). GEDI is a gestalt based algorithm that provides a global visual representation of gene expression profiles by transforming gene expression data into two-dimensional self-organizing maps by identifying pattern similarities [27]. GEDI analysis revealed the greatest differences in gene expression occurred 72 h after initial ANG II exposure, so we focused our analysis on these clusters of genes (Fig 2B). Natriuretic peptide B (*Nppb*), connective tissue growth factor, Rho family GTPase protein 1, and T-type calcium channel (*Cacna1g*, *Cacna1h*) genes were significantly upregulated in ANG II treated tissues at 72 h post initial ANG II exposure (Fig 2C–2F). These genes have been shown to be upregulated in response to ANG II exposure and are also upregulated in hypertrophy and heart failure in *in vivo* and *in vitro* studies [28–31]. ANG II exposure also led to a downregulation in T-box5 (*Tbx5*) and sodium channel (*Scna5*) gene expression levels, genes critical for proper function of the ventricular conduction system (Fig 2F and 2G) [32, 33]. These results show that ANG II induces a pathological gene expression profile in our engineered cardiac tissues.

### Angiotensin II increases probability for early after depolarizations

**Fig 3:**
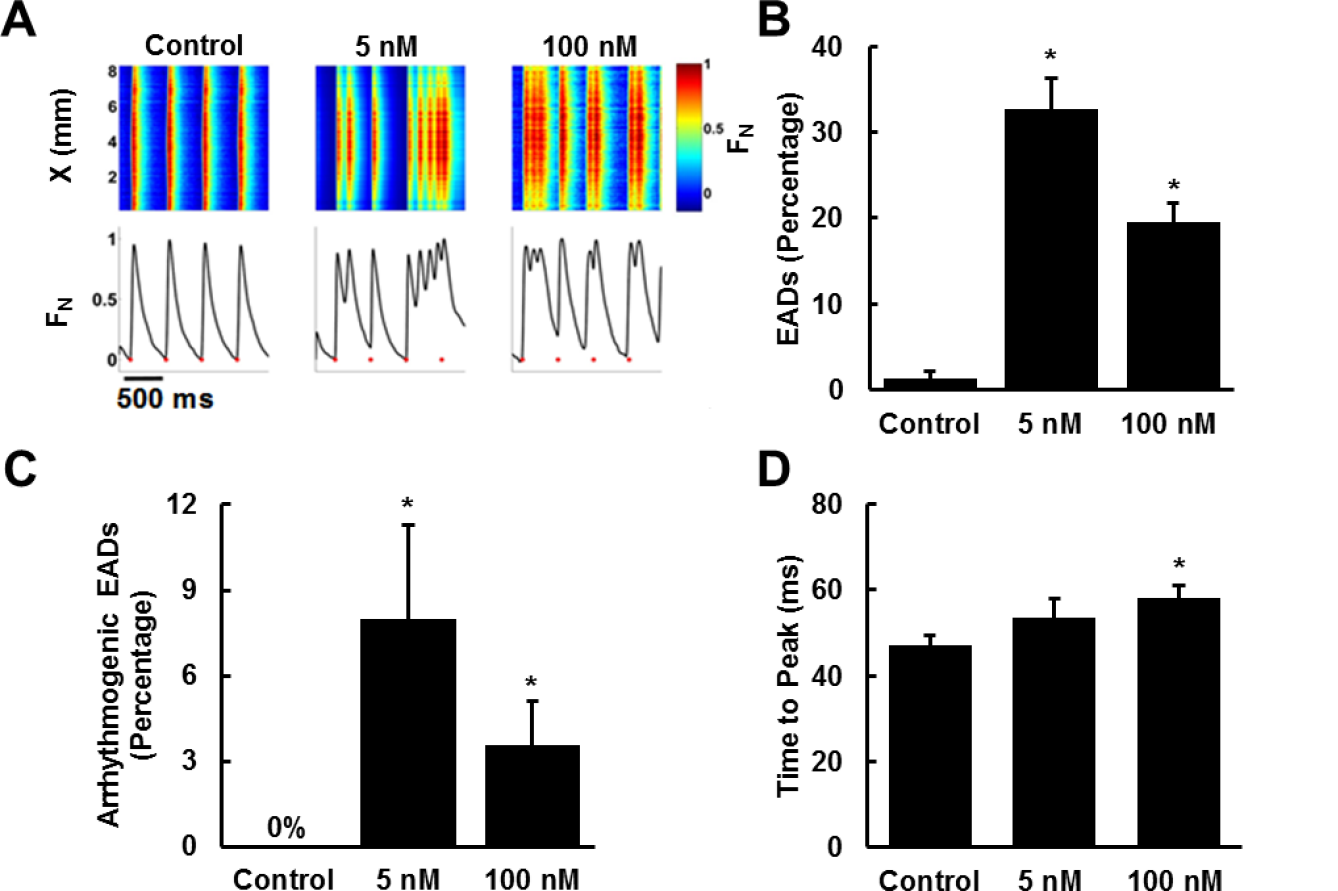
ANG II increases the occurrence of early after depolarizations (EADs) and arrhythmias. (A) Normalized fluorescence intensity of Rohd 2 dye (top panel) over time (*x*-axis) along a line crossing the 2D field of view (*y*-axis). Average fluorescence intensity traces (Bottom), red markers indicate the applied electrical pacing at 2Hz (Scale bar: 500 ms). Traces were normalized relative to the maximum peak intensity, F_N_ **=** F_instantaneous_/ F_max_. (B) Percentage EAD events relative to total paced events. (C) Percentage of EADs which evolved into sustained arrhythmia. (D) Time to peak for each condition. Control n = 8 tissues, 5 nM n = 8 tissues, 100 nM n = 10 tissues (mean ± SEM; * indicates p < 0.05 vs. control).

ANG II is known to induce proarrhythmic effects via several mechanisms [34–36] such as slowed conduction velocity and diminished cell coupling that facilitate reentrant pathways [37]. Specifically, ANG II has been shown to induce early after depolarizations (EADs) [37–39]. EADs are abnormal depolarizations that occur before normal repolarization is completed. These depolarization events may result from high intracellular calcium concentrations that induce premature release of calcium from the sarcoplasmic reticulum before repolarization completes [40, 41]. We asked if we could replicate ANG II induced arrhythmogenesis and pathological calcium transient properties in our model. Calcium transients in tissues paced at 1–2 Hz were imaged and analyzed for EADs and arrhythmias using Rhod2 dye (Fig 3A). ANG II significantly increased the probability of EADs relative to control tissues (Fig 3B). ANG II treatment also led to a significant increase in sustained arrhythmias, observed as either reentry cycles or tachyarrhythmic events whereas, control tissues generated no sustained arrhythmias during pacing (Fig 3C). These results suggest that we can recapitulate the proarrhythmic effects of ANG II in engineered cardiac tissues.

Calcium transient durations and calcium transient peak times increase during hypertrophy and heart failure [9]. We asked whether ANG II exposure would induce maladaptive changes in calcium transient morphology and assessed changes by quantifying the time to peak of calcium transients and calcium transient duration. ANG II treated tissues exhibited slight increases in calcium wave time to peak, with the 100 nM tissues exhibiting statistically significant time increases relative to control tissues (Fig 3D). These results suggest that ANG II induces modest remodeling of calcium transients and coupled with the proarrhythmic effects observed may result in contractile dysfunction.

### Angiotensin II leads to a decline in contractile function but only minimally influences tissue morphology

**Fig 4:**
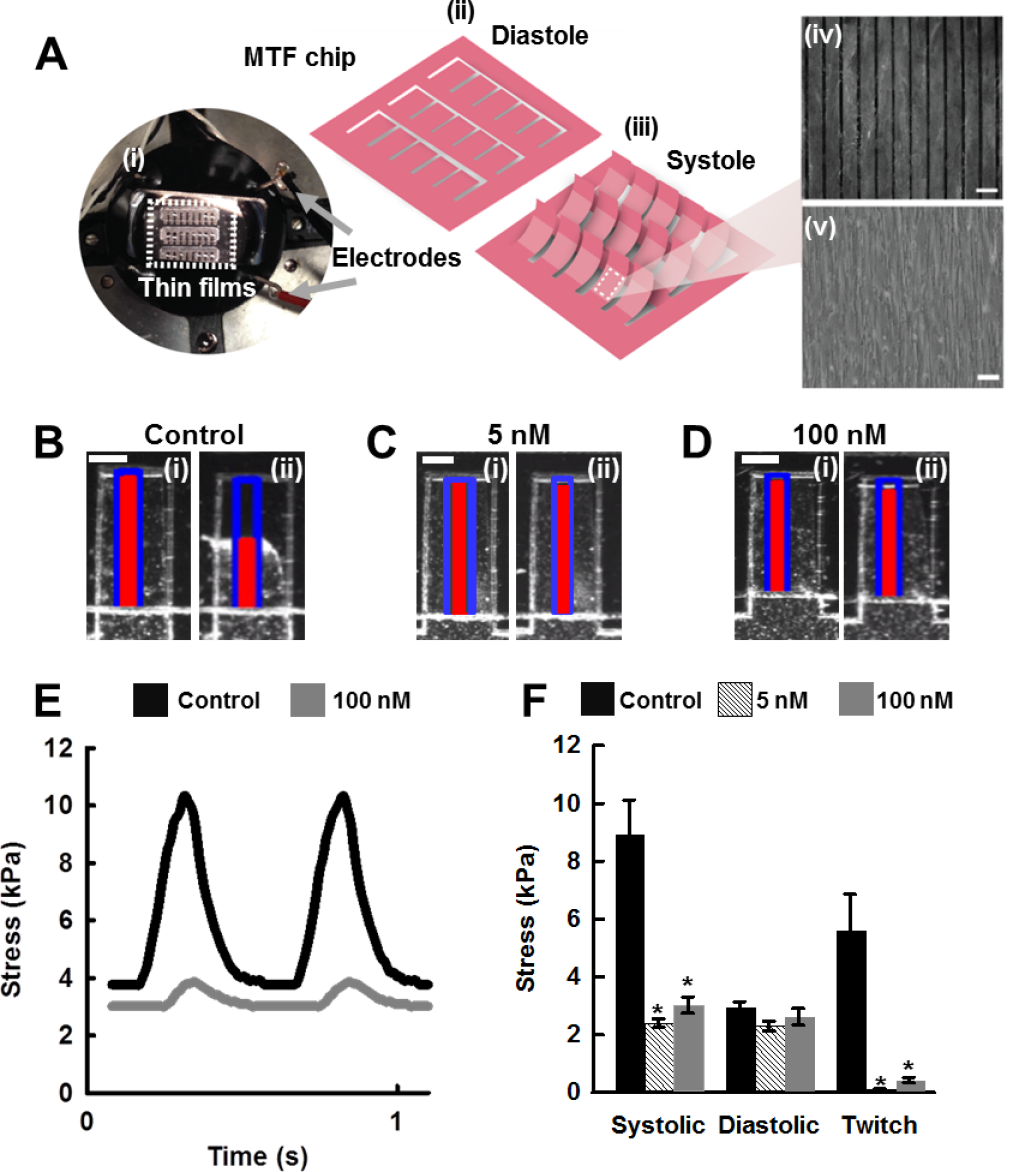
ANG II treatment leads to decreased contractile function. (A,) MTF Chip. (A,i) Photograph of experimental setup (Scale bar: 1 cm). (A ii-iii) Schematic of the MTF chip in diastole (ii) and systole positions (iii). Microcontact printing was used to pattern cells to resemble the structure of heart tissue architecture mimicked on the MTF chip (Scale bar: 20 μm). (A,iv) Fibronectin patterns on surface of thin film drive tissue architecture (Scale bar: 20 μm). (A,v) Brightfield image of engineered cardiac tissues on the MTF. (B-D) (i) Diastole and (ii) peak systole of muscular thin films. Blue outline represents the original film length, red line represents the x-projection of the radius of curvature of the film (Scale bar: 500 μm). (E) Representative stress traces generated from x-projections of films (F) ANG II treatment leads to a decrease in contractile stress generation. Control n = 85 tissues, 5 nM n = 15 tissues, 100 nM n = 69 tissues, 3 harvests. Tissues were paced at 2Hz (mean ± SEM, * indicates p < 0.05 vs control, # indicates p < 0.05 vs. 100 nM).

Elevated levels of intra-cardiac ANG II have been linked to reduced systolic pressure in transgenic mice that overexpress ANG II [16]. Thus, we asked whether ANG II induced contractile dysfunction could be measured using the MTF chip. After four days of ANG II treatment in culture, contractile studies were conducted. The systolic and diastolic positions of the MTFs were tracked and recorded (Fig 4A). These changes in film curvature resulting from tissue contractions were then converted into systolic stress generation and diastolic tension measurements using a modified Stoney’s equation [25]. The film displacement generated by treated and untreated tissues paced at 2 Hz were recorded (Fig 4B–4D) and subsequently converted into stress traces (Fig 4E) to assess ANG II effects on contractile function [25].

We observed an ANG II-induced decline in peak systolic stress generation relative to untreated tissues (Fig 4F). Twitch stress generation, defined as the difference between the peak systolic stress and the diastolic stress, was significantly attenuated in ANG II treated tissues relative to the control tissues. Interestingly, the 5 nM ANG II treated tissues exhibited greater functional degeneration than the 100 nM ANG II treated tissues (Fig 4F). There were no significant differences observed in diastolic stress generation between treated and control tissues. In addition to contractile effects, we asked if ANG II exposure induces structural remodeling within our system. We found that ANG II exposure led to marginal cell elongation in the 100 nM ANG II tissues exhibiting a statistically significant increase in aspect ratio relative to control tissues, 8.6:1 to 7:1, respectively (S1A Fig). However, ANG II exposure did not affect cell area (S1B Fig) or tissue thickness (S1C and S1D Fig) in our system. We further questioned whether ANG II exposure would alter sarcomere organization. Results revealed no significant differences in sarcomere organization between the treated and untreated tissues during our four day observation period (S1E Fig). Thus, we demonstrate that we can recapitulate and detect ANG II induced cardiac dysfunction in our *in vitro* model of heart failure which precedes structural remodeling.

## Discussion

ANG II contributes to the pathophysiology of cardiovascular diseases including vascular thickening, atherosclerosis, pathophysiological cardiac hypertrophy and remodeling, and ultimately heart failure [11, 42]. We have modeled ANG II induced cardiac dysfunction on a chip using engineered cardiac tissues that recapitulate the anisotropic, laminar structure of ventricular tissue. Gene expression profiles in ANG II treated tissues suggest a maladaptive state with the upregulation of T-type calcium channel genes, Rho family genes and ECM related genes which is consistent with *in vivo* and *in vitro* reports examining the hypertrophic effects of ANG II. ANG II treatment also led to increased EADs and conditions that potentiate arrhythmias and contractile failure. Taken together, these results suggest that we have created an *in vitro* cardiac dysfunction model that exhibits genetic and functional features of ANG II-induced failure for disease studies.

### ANG II and calcium handling

One of the hallmarks of hypertrophy and heart failure is the re-activation of the fetal gene profile which includes the re-expression of T-type calcium channel genes and a decrease in conduction related gene expression [43, 44]. Elevated levels of T-type calcium channels and the reappearance of T-type calcium currents have been observed in post-infarct and hypertrophied hearts and have also been associated with cardiomyopathies [45–47]. ANG II mediated increases in T-type calcium channel currents have been linked to cardiopathogenesis via calcium overload and arrhythmias [48]. Similarly, we observed an increase in T-type calcium channel gene expression in response to ANG II exposure which was accompanied by EADs and arrhythmic events in our system. ANG II treated tissues also exhibited a decline in T-box5 and *Scn5a* expression, factors critical for cardiac conduction. Previous studies show that ANG II exposure led to downregulation of T-box5, a transcription factor critical for proper heart development, and *Scn5a*, the gene that encodes for cardiac sodium channel Na_V_1.5, relative to control tissues. T-box5 and *Scn5a* are critical for ventricular cardiac conduction function [32]. Others have also reported that ANG II exposure resulted in the downregulation of *Scn5a* mRNA levels in H9c2 rat cardiomyocytes and suggested that ANG II may play a role in arrhythmogenesis by altering *Scn5a* transcriptional regulation through a nuclear factor kappa B (NF-κB) and oxidative stress mediated mechanism [49]. Cardiomyocytes containing a *Scn5a* mutation exhibited EADs [50]. Furthermore, genetic mutations of T-box5 and *Scn5a* have been linked to dilated cardiomyopathies and arrhythmias [32, 33] suggesting that these genes play a critical role in cardiac conduction [32, 51, 52]. These coinciding results, gene expression and calcium handling, suggest that ANG II treatment leads to conduction interruptions evident in the increased occurrences of EADs and arrhythmias in ANG II treated tissues relative to untreated tissues which may contribute to cardiac dysfunction.

### ANG II and contractile function

The electrophysiological remodeling that occurs in cardiac diseases contributes to arrthythmia development, systolic, and diastolic dysfunction which increase the risk of cardiac failure [48]. Contractile dysfunction is accompanied by changes in calcium transients. Interestingly, there were no major changes in calcium transients or durations even though contractile dysfunction was achieved in our system, a phenomenon observed by others [53]. Previous studies suggest that ANG II promotes activation of the p38 MAPK pathway and protein kinase C-β, a mediator of ANG II contractile effects, causing a reduction in myofilament sensitivity to calcium and ultimately resulting in a negative inotropic effect [53, 54]. In addition to cardiac conduction, ANG II mediated regulation of T-box5 has also been linked to cardiac dysfunction. T-box5 expression was downregulated in mice receiving ANG II infusions resulting in impaired fractional shortening [55]. Although we and others report that ANG II induces negative inotropic effects in cardiomyocytes [56–58] (rat, mouse, human), the effects of ANG II on contractility remain a debate as a range of responses have been reported. ANG II has also been reported to have no effect [59] (guinea pig infarct hearts) and positive inotropic effects [60, 61] (rabbit, cat) in other systems. These diverse results suggest that ANG II effects may vary between species and experimental parameters but also indicate that ANG II may be involved in a number of pathways that influence contractility. We also observed greater functional decline evident in statistically lower twitch stresses in the 5 nM ANG II group relative to the 100 nM ANG II treated group which may indicate that cells become desensitized to ANG II stimulation with higher concentrations. ANG II exposure has been shown to decrease cell responsiveness [62]. Previous studies using bovine adrenal glomerulosa cells show that longer exposures and higher concentrations corresponded with decreased receptor binding capacity [62]. Combined with our results, extended ANG II exposure at higher concentrations reduces cell responsiveness leading to attenuated functional effects in our cardiac tissues. Although, we observed decreased systolic and twitch stress generation in ANG II treated tissues, diastolic stress generation was not significantly different between groups. This result may be the consequence of elevated tissue fibrosis, a property of pathological hypertrophy as well as heart failure [63], which may lead to stiffer tissues thus, hindering cardiomyocyte relaxation which may be critical for diastolic dysfunction. We were also unable to resolve differences in relaxation times resulting from ANG II exposure implying that the use of PDMS limits our ability to distinguish PDMS recoil from the relaxation rate of the engineered cardiac tissues. We are currently exploring the use of other polymers and hydrogels to address system limitations.

In cardiac pathologies, the heart undergoes tissue-level and cellular level remodeling followed by functional failure. This remodeling is evident in ventricular wall thickening or thinning, fibrosis, cell shape changes, and cytoskeletal reorganization [64–66]. Previously, we modeled the failing myocardium using a dynamic system to mechanically induce maladaptive structural remodeling and cardiac dysfunction in engineered cardiac tissues via cyclic stretch [5]. Here we use similar microcontact printing techniques under static conditions and show that we can effectively isolate structural and functional remodeling, inducing cardiac dysfunction while maintaining the engineered structural features in our tissues. Insufficient paracrine signaling from cardiac fibroblasts may also play a role in the conserved tissue structure. Cardiomyocyte cultures containing little or no fibroblast populations exhibited modest or no cell growth effects in response to ANG II, respectively [23, 67]. Thus, we can infer that the fibroblast content in our system (approximately 5–10%) [68] falls below the minimum population required to adequately induce a hypertrophic morphology. In addition to system factors, the anti-growth effects of the AT_2_ receptor which counter the hypertrophic effects of the AT_1_ receptor may mitigate cell growth in response to ANG II [69]. We assert that our system design and ANG II receptor properties attribute to the conserved architecture of our engineered tissues. Regardless, this system is unique in that we can probe genetic and functional changes resulting from ANG II exposure independent of significant structural remodeling.

**Fig 5:**
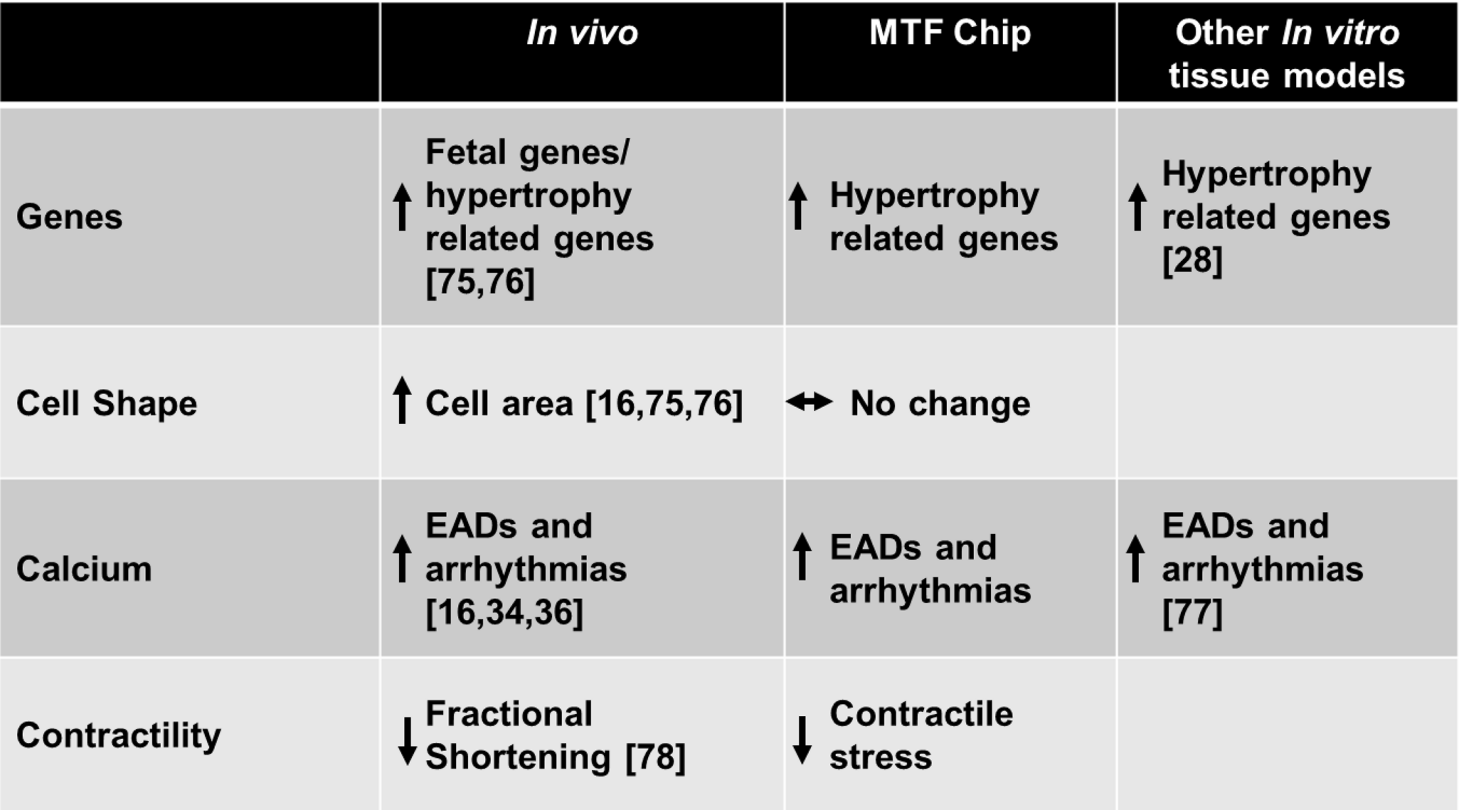
MTF chip performance assessment. Results from this study were used to compare the MTF chip based model to *in vivo/ex vivo* and other *in vitro* tissue model studies. Many models only examine certain features of pathology, this MTF chip based model mimics ventricular tissue architecture and generates multiscale readouts and can be used to compare healthy and diseased tissues.

We assert that *in vitro* disease models using the heart-on-a-chip platform can serve as a suitable platform for disease mechanism studies and pharmaceutical applications. In this study, we created an ANG II induced cardiac dysfunction model capable of recapitulating pathological genetic profiles, calcium behavior, and cardiac dysfunction indicative of cardiac pathophysiology while conserving structural architecture. Although this study highlights engineered cardiac tissues composed primarily of cardiomyocytes, the heart consists of fibroblasts and endothelial cells which play an integral role in organ structure and various paracrine signaling cascades. We are currently developing co-culture models to address this limitation. Our model complements assays that use single cell and animal models, offering the ability to perform specific studies on isolated cell types while recapitulating native heart tissues structure in a simplified platform. The ANG II cardiac dysfunction on a chip model mimics many features of disease observed *in vivo/ ex vivo* and offers the ability to constrain tissue architecture to independently examine ANG II effects on cardiac tissue function (Fig 5). Taken together, our findings highlight the robust, multiscale analytical capabilities of our MTF chip for characterizing healthy and diseased tissues.

## Materials and Methods

### Ethics Statement

All procedures used in this study were approved by the Harvard Animal Care and Use Committee under Animal Experimentation Protocol permit number 24–01 entitled “Harvest and Culture of Neural and Cardiac Tissue from Neonatal Rats and Mice for *In Vitro* Disease Models”. This protocol meets the guidelines of the Faculty of Arts and Sciences of Harvard University pertaining to the use of vertebrate animals in research and teaching. This protocol also follows recommendations included in the NIH Guide for the care and use of laboratory animals and is in accordance with existing Federal (9 CFR Parts 1,2 &3), state and city laws and regulations governing the use of animals in research and teaching.

### Muscular thin film fabrication

We have previously reported the MTF fabrication technique was used for this study [24]. Briefly, poly(NIPAAm) (Polysciences, Inc., Warrington, PA) islands, the sacrificial layer, were spun coated onto glass coverslips (18 mm x 18 mm). The glass coverslips were then coated with Sylgard 184 polydimethylsiloxane (PDMS) (Dow Corning, Midland, MI) and cured at 65°C overnight. Arrays of rectangular thin films (3 mm long x 1 mm wide) were then cut into the PDMS layer using an Epilog CO_2_ laser (Epilog Laser, Golden, CO). The film thickness was determined using a contact Veeco profilometer (Dektak 6M, Veeco Instruments Inc., Plainview, NY). Film thickness ranged between 11–13 μm.

### Microcontact printing

Engineered cardiac tissues were created by seeding cardiomyocytes onto microcontact printed templates [68, 70, 71]. Briefly, a mask containing rectangular arrays of 15 μm lines with 2 μm spacings was created using AutoCad (Autodesk, Inc., San Rafael, CA). Silicon wafers coated with SU-8 2002 photoresist (MicroChem, Newton, MA) were exposed to UV light followed by SU-8 developer to resolve the exposed mask features. The wafer then underwent salinization and stamps were created by pouring PDMS over the wafer. After degassing, the PDMS was allowed to cure at 65°C for at least 4 h. The cured PDMS stamps were then cut from the wafer. PDMS stamps containing an array of 15 μm wide lines with 2 μm spaces were used to pattern fibronectin onto PDMS coated surfaces. Microcontact printing was performed by “inking” PDMS stamps with 50 μg/mL fibronectin (BD Biosciences, Bedford, MA) for 1 h. The stamps were then dried and inverted parallel to the direction of UV ozone treated PDMS thin films. The chip was then background treated with a 2.5 μg/ml concentration of fibronectin for 15 min and rinsed three times with PBS. For the gene expression samples, chips were treated with 1% Pluronics (BASF, Florham Park, NJ) to prevent non-specific cell binding. Cells were then seeded at a concentration of 100,000 cells/cm^2^.

### Cell harvest

All animal protocols were approved by the Harvard University Animal Care and Use Committee. All materials were purchased from Invitrogen (Carlsbad, CA) unless otherwise stated. A previously published cardiomyocyte harvest protocol was used for this study [68, 70, 71]. Briefly, two day old Sprague-Dawley rats (Charles River Laboratories, Inc., Wilmington, MA) were sacrificed via decapitation. Ventricles were harvested, rinsed with Hank’s Balanced Salt Solution, minced, and enzymatically digested using 1 mg/ml trypsin (USB Corp., Santa Clara, CA) for 12 h at 4°C. Tissues underwent four mechanical dissociations steps in 1mg/mL collagenase II (Worthington Biochemical, Lakewood, NJ) to isolate single cells. In order to reduce the fibroblast population, two 45 min preplating steps were incorporated. During the preplating step, the cell suspension was incubated in a flask containing M199 media supplemented with 10% fetal bovine serum (FBS) for 45 min at 37°C. The cell suspension was then collected and transferred to a fresh flask containing M199 media supplemented with 10% FBS for the second preplate step. Again, the cell suspension was collected and cells were counted. Cells were then seeded onto chips at a density of 100,000 cells/cm^2^. Cardiomyocytes were cultured in media composed of M199 basal media supplemented with 10% FBS, 2 mM L-glutamine, 0.1 mM nonessential amino acids, 10 μM HEPES, 19.4 μM glucose (Sigma Aldrich, St. Louis, MO), 50 U/mL of penicillin, and 1.5 μM B12 (Sigma Aldrich, St. Louis, MO) for the first 48 h. Cells were cultured in M199 media supplemented with 0.25% BSA (Sigma Aldrich, St. Louis, MO) rather than FBS for the remaining 48 h. Media was changed daily. Treated tissues were exposed to 5 nM or 100 nM ANG II (Sigma Aldrich, St. Louis, MO) dissolved in water daily at 24 h, 48 h, and 72 h post plating.

### Gene expression

Cell lysates were collected at 6 h, 24 h, and 72 h after initial ANG II dosing. RNA was extracted using the Stratgene Absolutely RNA Miniprep kit (Agilent Technologies, Santa Clara, CA). RNA concentration was determined using the Nanodrop spectrophotometer (Thermo Scientific, Waltham, MA). A first strand cDNA kit (Qiagen, Valencia, CA) was used to convert 500 ng of RNA to cDNA. A total of 90 genes (S1–S3 Tables) were examined using a custom PCR array (Qiagen, Valencia, CA). Gene expression was then quantified using the Bio-Rad CFX96 real-time PCR detection system (Bio-Rad, Hercules, CA). The Gene Expression Dynamic Inspector (GEDI) program was used to create gene clusters [27, 72]. GEDI uses machine learning algorithms to identify genes that exhibit similar expression profiles. These clusters were then organized into a 5x4 gene tile grid with each tile containing genes with similar expression profiles. Tile colors were assigned based on the centroid value for the gene clusters indicating upregulated or downregulated genes and the color cutoff limit was set at three standard deviations from the mean. Gene expression was normalized to untreated tissues at respective timepoints (6 h, 24 h, and 72 h post ANG II exposure). Relative fold change and fold regulation were determined using the ΔΔCt method [73]. Fold regulation is defined as the negative inverse of the fold change when the fold change value is less than one and fold regulation is equivalent to fold change when the fold change value is one or greater than one. The heatmap was generated using the statistical computing R program (http://www.r-project.org) and scaled based on column Z scores. The Z scores were calculated by subtracting the mean value (computed across all clustered samples) from each fold regulation value and divided by the corresponding standard deviation using the R program. Samples and genes were then clustered based on similarities.

### Sarcomeric organization analysis

Reagents were purchased from Invitrogen (Carlsbad, CA) unless otherwise noted. Tissues were fixed with 4% paraformaldehyde (Electron Microscopy Sciences, Hatfield, PA) for 15 min and stained for monoclonal mouse sarcomeric α-actinin (Sigma-Aldrich, St. Louis, MO) and polyclonal rabbit fibronectin (Sigma-Aldrich, St. Louis, MO) for 2 h at room temperature. Tissues were then rinsed multiple times with PBS and incubated with Alexa Fluor 488 goat anti-mouse IgG, Alexa Fluor 546 goat anti-rabbit IgG, Alexa Fluor 633 phalloidin, and 6-diamidino-2-phenylindole (DAPI) for 1 h at room temperature. A 1:200 dilution in PBS was used for all antibodies and chemical stains. Samples were rinsed with PBS, coated with ProLong Gold Anti-Fade reagent, mounted on a glass slide, and sealed with clear nail polish (Electron Microscopy Sciences, Hatfield, PA). Tissues were then imaged on a Leica SP5 X MP Inverted Laser Scanning Confocal Microscope (Leica, Wetzlar, Germany) with a 63x glycerin objective. A total of 9–10 fields of view were imaged per sample. Sarcomere analysis was performed using a custom ImageJ (NIH, Bethesda, MD) macro. Briefly, confocal stacks were converted into 2D images using a maximum *z*-projection. Using the structure tensor algorithm, orientation and coherency were detected pixel-wise. The global orientation order parameter (OOP) was then calculated using the orientations detected from pixels that had coherency and intensity values >30% of the maximum, thus filtering out spurious detection [25, 68, 74].

### Tissue thickness analysis

Fixed cardiac tissues were incubated with Cell Mask® (Invitrogen, Carlsbad, CA) for 10 min. The tissues were rinsed three times with PBS (Invitrogen, Carlsbad, CA), mounted and sealed as described above, and imaged on a Leica SP5 X MP Inverted Laser Scanning Confocal Microscope (Leica, Wetzlar, Germany) with a 63x glycerin objective. A total of 9 fields of view were imaged per sample. Images were rotated 90° in the *x* direction and confocal stacks were converted into 2D images using a sum *z*-projection. The resulting image was then subjected to a threshold, converted to a binary image, and tissue thickness was measured by outlining the tissue layer to obtain width measurements using ImageJ (NIH, Bethesda, MD).

### Cell shape analysis

Cardiac tissues were incubated with 10 μM di-8-ANEPPS (Invitrogen, Carlsbad, CA) for 15 min at 37°C to stain the cell membrane. Tissues were then rinsed with normal Tyrode’s solution (1.8 mM CaCl_2_, 5 mM glucose, 5 mM HEPES, 1 mM MgCl_2_, 5.4 mM KCl, 135 mM NaCl, 0.33 mM NaH_2_PO_4_, pH 7.4) (Sigma Aldrich, St. Louis, MO) and imaged at 37°C using a Leica SP5 X MP Inverted Laser Scanning Confocal Microscope (Leica, Wetzlar, Germany) with a 63x glycerin objective. The cell borders were traced manually in ImageJ (NIH, Bethesda, MD), the cell area was then measured by fitting the traced area to an ellipse, and the aspect ratio was determined using the ratio of the major axis (length of cell) to the minor axis (width of cell).

### Calcium studies

Cardiac tissues were incubated with 2 μM Rhod2® (Invitrogen, Carlsbad, CA) for 30 min at 37°C, rinsed, and incubated in dye free media for an additional 15 min. Tissues were then rinsed with 1x Normal Tyrode’s buffer and transferred to a heating stage. The engineered cardiac tissues were allowed to equilibrate for 5 min prior to recording calcium fluorescence. The calcium fluorescence signal was captured with a MiCAM ULTIMA high speed camera (SciMedia, Costa Mesa, CA) at a frame rate of 1 kHz with a field of view of 10 mm x10 mm, centered relative to the sample in the petri dish. Calcium fluorescence was detected and recorded from a quarter of the entire sample area at a time using a Tandem-Lens microscope (SciMedia, Costa Mesa, CA) with a Leica plan APO 1x objective (Leica, Wetzlar, Germany). Calcium fluorescence was recorded during both spontaneous and stimulated activity. For stimulated activity, cardiac tissues were paced at 1–2 Hz using a Myopacer field stimulator (IonOptix Corp., Milton, MA).

A custom Matlab (Mathworks, Natick, MA) peak detection algorithm was used to detect calcium waves. Calcium transients were normalized relative to the maximum peak intensity. The onset times and 90% repolarization times were detected for each of the stimulated calcium waves and were used to calculate time to peak and duration. Early after depolarizations (EADs) were counted and the probability for EAD was calculated as total EAD events out of the total number of electrical stimulated triggered activity. Additionally, the total number of arrhythmic events was also counted. Arrhythmic events were defined as either EADs that developed into a reentrant cycle or tachyarrhythmic events.

### Muscular thin film assay and analysis

Muscular thin films were placed in warm Tyrode’s solution and the temperature was allowed to briefly fall below 32°C to facilitate the phase transition of the sacrificial poly(NIPAAm) (Polysciences, Inc., Warrington, PA) layer to an aqueous phase allowing for easy release of the PDMS film from the glass base. Once the poly(NIPAAm) reached aqueous phase, films were gently peeled from the glass and tissues were paced at 2 Hz using a Myopacer field stimulator (IonOptix Corp., Milton, MA). The voltage was adjusted to 20% above the minimum voltage required to capture 90% of the total MTFs. Videos were recorded using a Basler camera at 100 frames per second for 3 seconds using Lab View (National Instruments, Woburn, MA) and imaged using a Zeiss Discovery V8 Stereo microscope (Oberkochen, Germany). All experiments were performed at 36 ± 1°C. Custom ImageJ and Matlab codes were created to calculate peak systolic, diastolic, and twitch stress generation using the film radius of curvature, measured PDMS film thickness, and cell layer thickness as inputs into a modified Stoney’s equation [25].

### Statistical analysis

A student’s *t*-test was performed with Microsoft Excel® (Microsoft, Redmond, WA) for cell shape, sarcomere organization, tissue thickness, calcium, and muscular thin film studies. *P* values of less than 0.05 or 0.01 were considered statistical significant and are noted appropriately. Two-way ANOVA analysis was performed with SigmaPlot (Systat Software, Inc., San Jose, CA) to determine statistical significance relative to control tissues for gene expression studies. Genes with *p* values less than or equal to 0.01 or less than 0.05 were considered significant and are also indicated appropriately.

## Conclusions

In this study, we probed whether we could recapitulate ANG II induced cardiac dysfunction on a chip using our heart-on-a-chip platform. We found that ANG II induced gene expression profiles indicative of cardiac dysfunction. This model is unique in that in the absence of appreciable structural remodeling, functional impairment was still achieved. ANG II tissues exhibited higher occurrences of EADs and arrhythmias. Furthermore, force measurements revealed that the treated tissues generated lower systolic stresses relative to the untreated controls. Observations from this study, supported by previously reported ANG II effects, confirm that we have effectively modeled cardiac dysfunction on a chip that recapitulates reported features of heart failure, presenting the MTF chip as a sensitive organ-on-chip platform for disease modeling. Results from this study could be used to design mechanistic studies to better characterize ANG II receptor contributions to physiological or pathological states and identify novel pathways for ANG II induced cardiac dysfunction using our MTF chip.

## Supporting Information

S1 Fig Angiotensin II has minimal effects on cell morphology. (A-B) Engineered cardiac tissues were stained with di-8-ANEPPS to delineate the cell borders within the tissues for aspect ratio analysis. Measurements indicate that ANG II (n = 8 tissues, mean ± SEM, p < 0.05 vs. control) does not alter cell shape or cell area. (C) Representative image of tissues were stained with a deep red cell mask dye and thickness was determined using a composite of Z-stack images. A threshold was applied to the image and the cell layer thickness was measured using ImageJ. (D) Angiotensin II has no effect on the thickness of tissues. (mean ± SEM, n = 4 tissues) (E) Tissues were stained for sarcomeric α-actinin to determine sarcomere organization. Sarcomere orientational order parameter (OOP) analysis shows that sarcomere alignment is not affected by ANG II exposure. (n = 6 tissues, mean ± SEM) (Scale bar: 10 μm).
(TIF)

S1 Table PCR Gene Array Primers. (TIF)

S2 Table PCR Gene Array Primers. (TIF)

S3 Table PCR Gene Array Primers. (TIF)
