# Supplementary figures and images for: Angiotensin II Induced Cardiac Dysfunction on a Chip
Source: PLoS One. 2016 Jan 25;11(1):e0146415. doi: 10.1371/journal.pone.0146415 (PMC4725954; doi:10.1371/journal.pone.0146415)

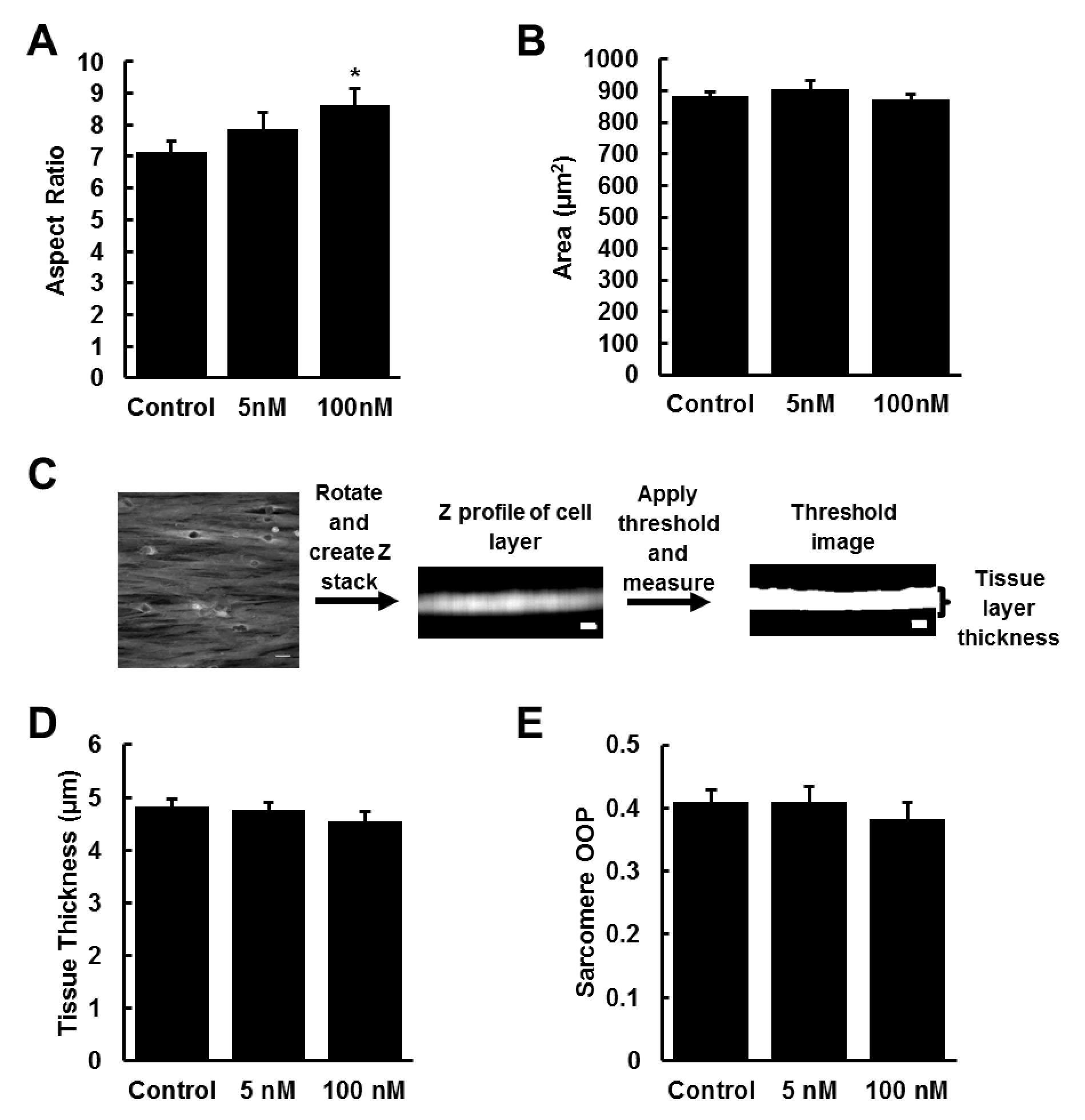

Supplement: S1 Fig — (A-B) Engineered cardiac tissues were stained with di-8-ANEPPS to delineate the cell borders within the tissues for aspect ratio analysis. Measurements indicate that ANG II (n = 8 tissues, mean ± SEM, p < 0.05 vs. control) does not alter cell shape or cell area. (C) Representative image of tissues were stained with a deep red cell mask dye and thickness was determined using a composite of Z-stack images. A threshold was applied to the image and the cell layer thickness was measured using ImageJ. (D) Angiotensin II has no effect on the thickness of tissues. (mean ± SEM, n = 4 tissues) (E) Tissues were stained for sarcomeric α-actinin to determine sarcomere organization. Sarcomere orientational order parameter (OOP) analysis shows that sarcomere alignment is not affected by ANG II exposure. (n = 6 tissues, mean ± SEM) (Scale bar: 10 μm). (TIF) [file pone.0146415.s001.tif]

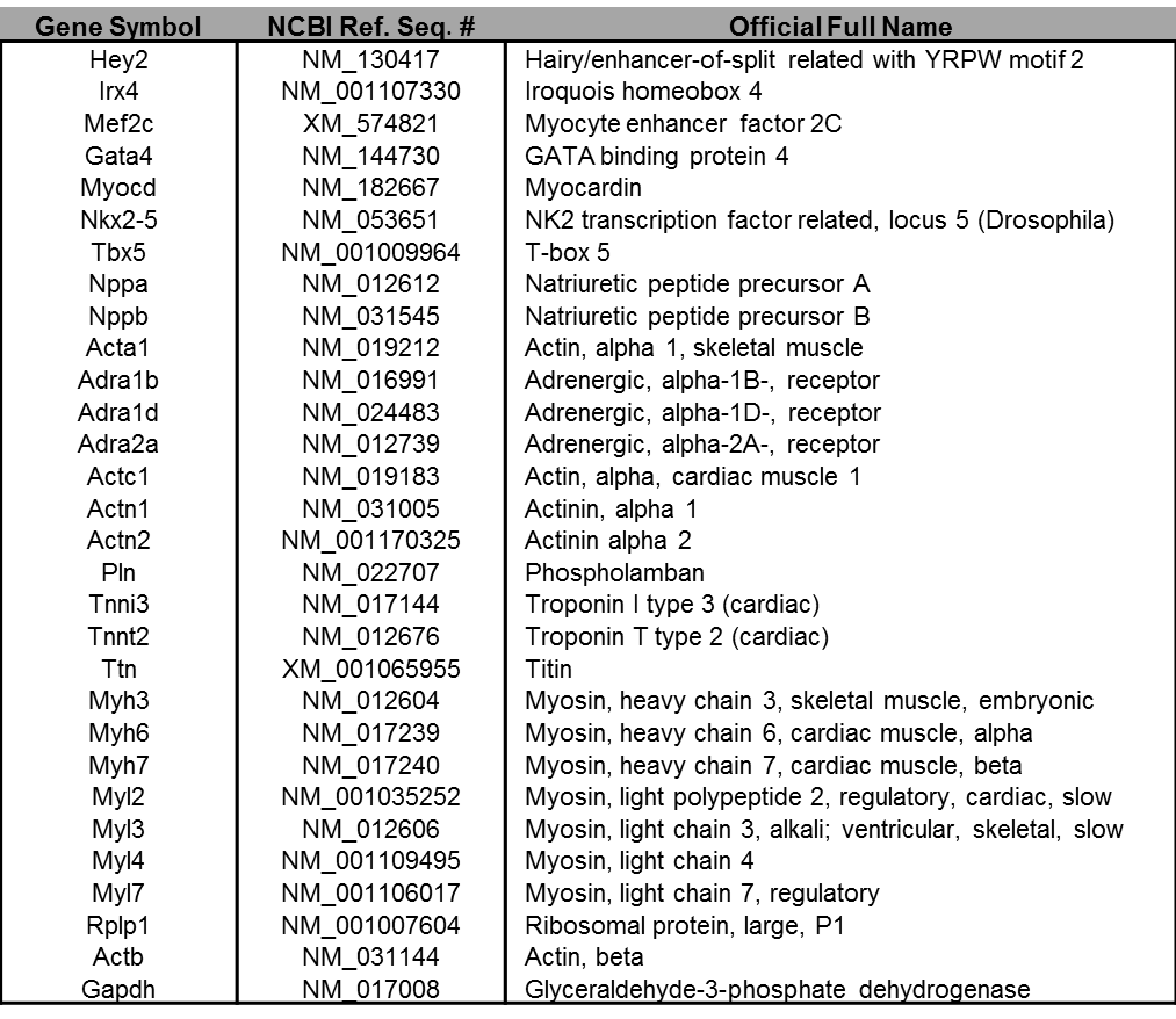

Supplement: S1 Table — (TIF) [file pone.0146415.s002.tif]

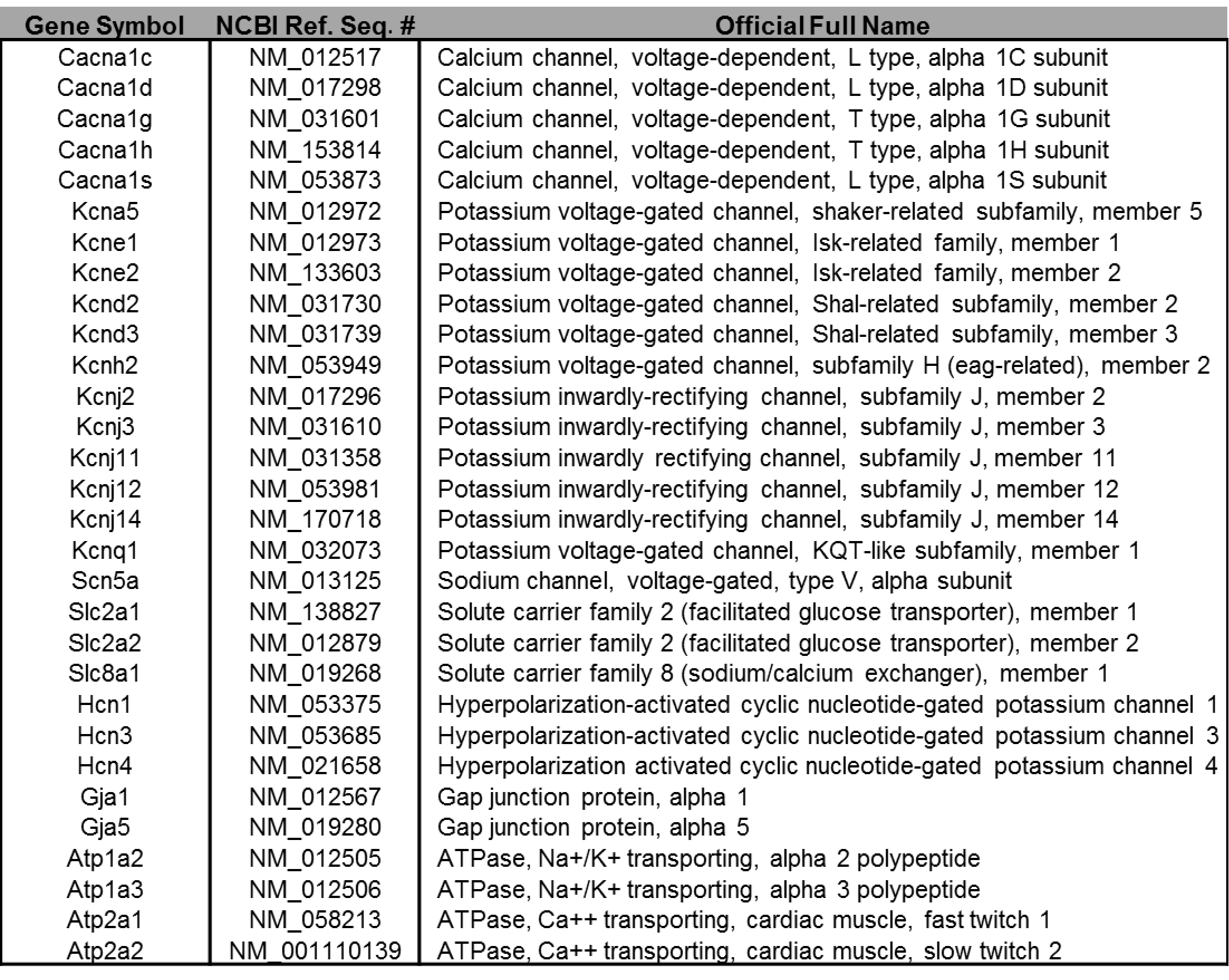

Supplement: S2 Table — (TIF) [file pone.0146415.s003.tif]

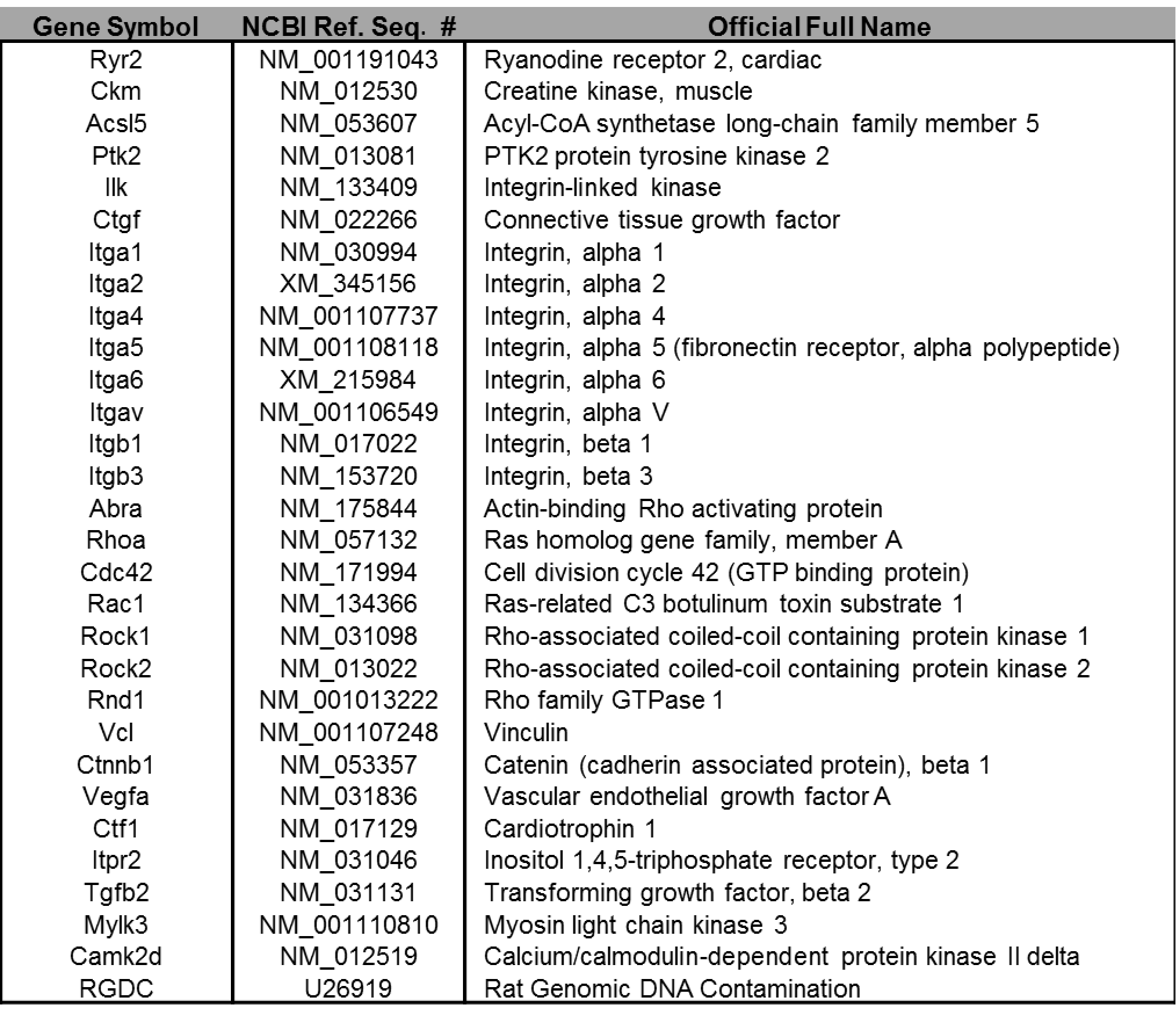

Supplement: S3 Table — (TIF) [file pone.0146415.s004.tif]
